# Supplementary material for: Training-free CryoET Tomogram Segmentation
Source: ArXiv. 2024 Jul 8:arXiv:2407.06833v1. Preprint. [Version 1] (PMC11261984)
Supplement: Supplement 1 [file NIHPP2407.06833v1-supplement-1.pdf]

Supplementary Material

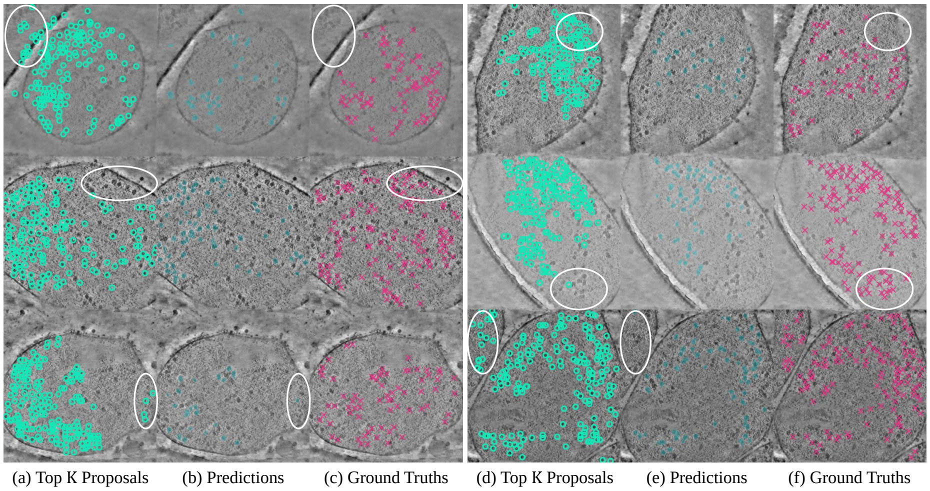

Fig. 6. Failure cases of CryoSAM. 1st row: False Positive in proposed prompts. 2nd row: False Negative in proposed prompts. 3rd row: False Negative in final predictions.

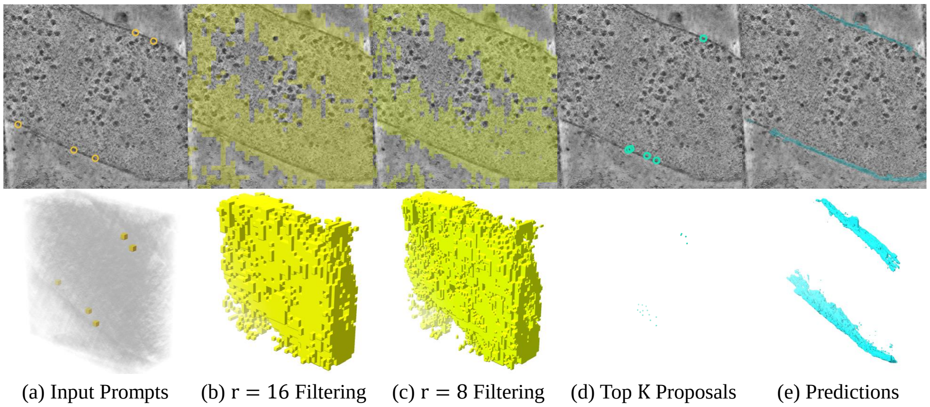

Fig. 7. Intermediate and final predictions of CryoSAM for membrane segmentation. CryoSAM can segment membranes with sparse prompt inputs.

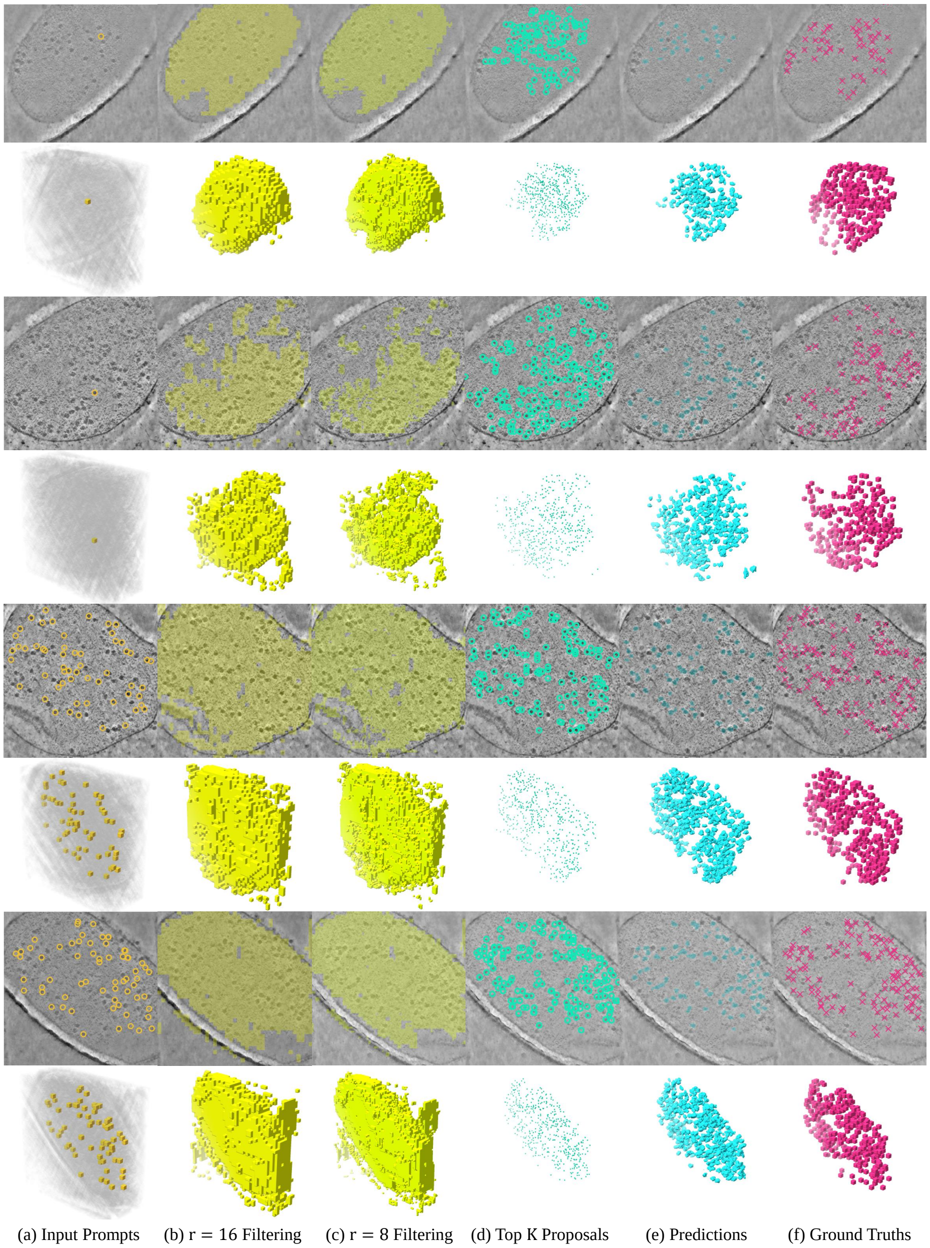

Fig. 8. Intermediate and final predictions of CryoSAM for particle picking. We provide additional results for feeding CryoSAM with both single-point prompts and multiple-point prompts. In columns (a), (d), and (f), we show points with coordinates ranging from $z - 20$ to $z + 20$ for demonstration, where z is the coordinate of the visualized tomogram slice.
